# Evaluation of overwintering risk of tropical and subtropical insect pests in temperate regions

**DOI:** 10.1038/s41598-024-82713-z

**Published:** 2024-12-28

**Authors:** Keiichiro Matsukura, Nobuo Mizutani, Sayumi Tanaka, Yoshiaki Tanaka

**Affiliations:** 1 Institute for Plant Protection, National Agriculture and Food Research Organization (NARO), Tsukuba, Ibaraki Japan; 2 Koshi Research Station, Institute for Plant Protection, NARO, Kumamoto, Koshi Japan; 3 Institute of Agrobiological Sciences, NARO, Tsukuba, Ibaraki Japan; 4 Division of Crop Pest Control Research, Institute for Plant Protection, NARO, Kannondai 2-1- 18, Tsukuba, 305-8666 Ibaraki Japan

**Keywords:** Biological invasion, Cold tolerance, Crop pest, Gridded climate data, Global warming, Species distribution model, Agroecology, Ecological modelling, Ecophysiology, Invasive species

## Abstract

Recent changes in climate and environments have promoted the range expansion of insect pests of tropical and subtropical origins into temperate regions. For more accurate and faster risk assessment of this expansion, we developed a novel indicator to link a physiologically derived parameter of chilling injury with the survival of insect populations in nature by using two insects, *Spodoptera frugiperda* and *Cicadulina bipunctata* with tropical and subtropical origins, and one cool-adapted insect, *Laodelphax striatellus*. The parameter derived from a proportional increment in the time to 99.9% mortality under constant low temperatures causing chilling injury evaluates the survival of target insect populations based on winter climate data. For *S. frugiperda* and *C. bipunctata*, but not for *L. striatellus*, the accuracy of the model in predicting the overwintering range was equivalent to, or better than, those of a conventional species distribution model. Additional field testing using *S. frugiperda* and comparison of the developed model with a conventional logistic model for *C. bipunctata* supported the validity of the indicator. These results suggest that the developed indicator will help for simple risk assessment of tropical and subtropical insect pests in temperate regions by the species distribution modelling approach.

**Supplementary Information:**

The online version contains supplementary material available at 10.1038/s41598-024-82713-z.

## Introduction

Winter climate is a critical factor restricting the distribution ranges of plants and animals in temperate and polar habitats^1,2^, because low temperatures during winter can cause physiological disorders that are often lethal^3,4^. Recent deterioration of this natural climatic barrier because of climate change is resulting in new invasions of temperate areas by organisms from warmer areas. These organisms can influence native ecosystems by, for example, altering the local abiotic and biotic environment^5,6^, causing the extinction or reduction of populations of beneficial native organisms^7^, or threatening food production by acting as crop pests or weeds^8,9^.

Predicting and evaluating the risk of range expansion of tropical and subtropical insects is important to protect crop production in temperate regions. A major approach to estimating present and future species distributions is the use of species distribution models (SDMs). These models evaluate the availability of each niche for target insects, mainly by using abiotic environmental factors. Correlative SDMs are traditional models that link observed presence and absence records of target insects with environmental variables by using statistical models. They were first developed from regression-based analyses^10^, but the recent preference is to use machine-learning techniques, as represented by maximum entropy and random forest models^11^. Despite their simple concept and applicability to a variety of organisms^12–14^, correlative SDMs often fall short in the ecological interpretation of their outputs. Correlative SDMs mathematically optimize the parameters so that the model explains the presence and absence records with minimum error. Therefore, the optimized parameters do not always ecologically involve the presence or absence of the target species^15^. Moreover, the model accuracy must be considered carefully when SDMs are applied to invasive species in their non-native ranges, because model parameters are usually optimized on the basis of presence and absence records and related environmental factors in a species’ native range^16^.

To overcome the shortfalls of correlative SDMs, mechanistic SDMs are becoming more popular^17^. Mechanistic SDMs consider the distribution of target organisms and niche availability by using physiological parameters such as development rate, fecundity, and cold and heat tolerance. Their use should therefore yield more biologically significant estimations than those from correlative SDMs, which associate presence/absence records with climate and environmental conditions. In particular, tolerance to cold injury is one of the most important qualities that allow invasive organisms originating from tropical and subtropical regions to survive and spread to temperate regions.

Cold injury is generally classified into two types, depending on whether the injuries occur from freezing or from chilling^18,19^. Freezing occurs at lower temperatures than chilling and is an important lethal factor, mainly in polar and cold regions^20^. On the other hand, chilling (i.e., non-freezing) injury caused by moderately low temperatures of around 0 °C or even higher is more important to surviving winter climates in temperate regions. The moderately low temperatures in this range rarely freeze organisms but induce physiological stresses, known as chilling injury, in the organisms’ bodies^21,22^. For insects of tropical and subtropical origins, both freezing and chilling injuries are obstacles to be overcome to survive winter in temperate regions.

Several physiological indicators have been developed to quantify the tolerance to low temperatures in insects. Supercooling point is a popular indicator to evaluate the freeze avoidance ability of insects^23^. Many insects that can not survive freezing keep their body fluids unfrozen even at temperatures below the equilibrium freezing point, by producing antifreeze proteins and low molecular weight cryoprotectants. These insects supposed to die when they are exposed to temperatures lower then their supercooling point, therefore, lower supercooling point simply means higher tolerance to low temperatures around freezing points. In evaluations of tolerance to chilling injury, several quantitative indicators such as the lethal time, critical thermal minimum, and chill coma temperature have been also proposed^24,25^. However, the quantitative relationship between these indicators and the survival of species in nature have yet been confirmed. This is because of insufficient understanding about the mechanism of chilling injury compared to those about freezing injury despite recent advances in the mechanism of chilling injury such as membrane phase transition, protein denaturation, and loss of ion homeostasis^21,22,26^.

In this study, we developed a novel indicator of tolerance to chilling injury that can be used for evaluating the overwintering risk of tropical and subtropical insects in temperate regions by SDM approach. The indicator was developed by focusing on the proportional increment of time required to kill 99.9% individuals of insect population (LT_99.9_) depending on the increment of temperature within a range causing chilling injury. The accuracy of the indicator was examined by using two tropical/subtropical insects and one temperate insect. The fall armyworm, *Spodoptera frugiperda*(Smith) (Lepidoptera: Noctuidae) is a lepidopteran pest of tropical and subtropical origin in the Western Hemisphere and has quickly expanded its distribution worldwide^27^. After the first invasion of temperate regions in Japan by *S. frugiperda*in 2019, it was found in a wide range from subtropical Southwestern Islands to subarctic regions of Hokkaido from spring to summer^28,29^, however, a geographic origin analysis by using Strontium isotope (i.e., 87Sr / 86Sr isotope ratio) supported the overwintering of this moth only in the Southwestern Islands^30^. The maize orange leafhopper, *Cicadulina bipunctata (Melichar)*(Hemiptera: Cicadellidae) is distributed in tropical and subtropical regions of the Old World^31^. This hemipteran pest expanded its distribution range to the plains areas at about latitude 33°N or lower in Japan in the early 2010s^32^under increased air temperatures in winter and early summer^33^. We also tested our model by using the small brown planthopper, *Laodelphax striatellus*(Fallén) (Hemiptera: Delphacidae) to examine an applicability of our model for insects originating in colder regions. This planthopper did not originate in tropical regions but has been distributed over a wide range from subtropical to subarctic regions in East Asia^34,35^.

## Results

### Linear change of LT99.9 with temperature and parameter determination

**Fig. 1:**
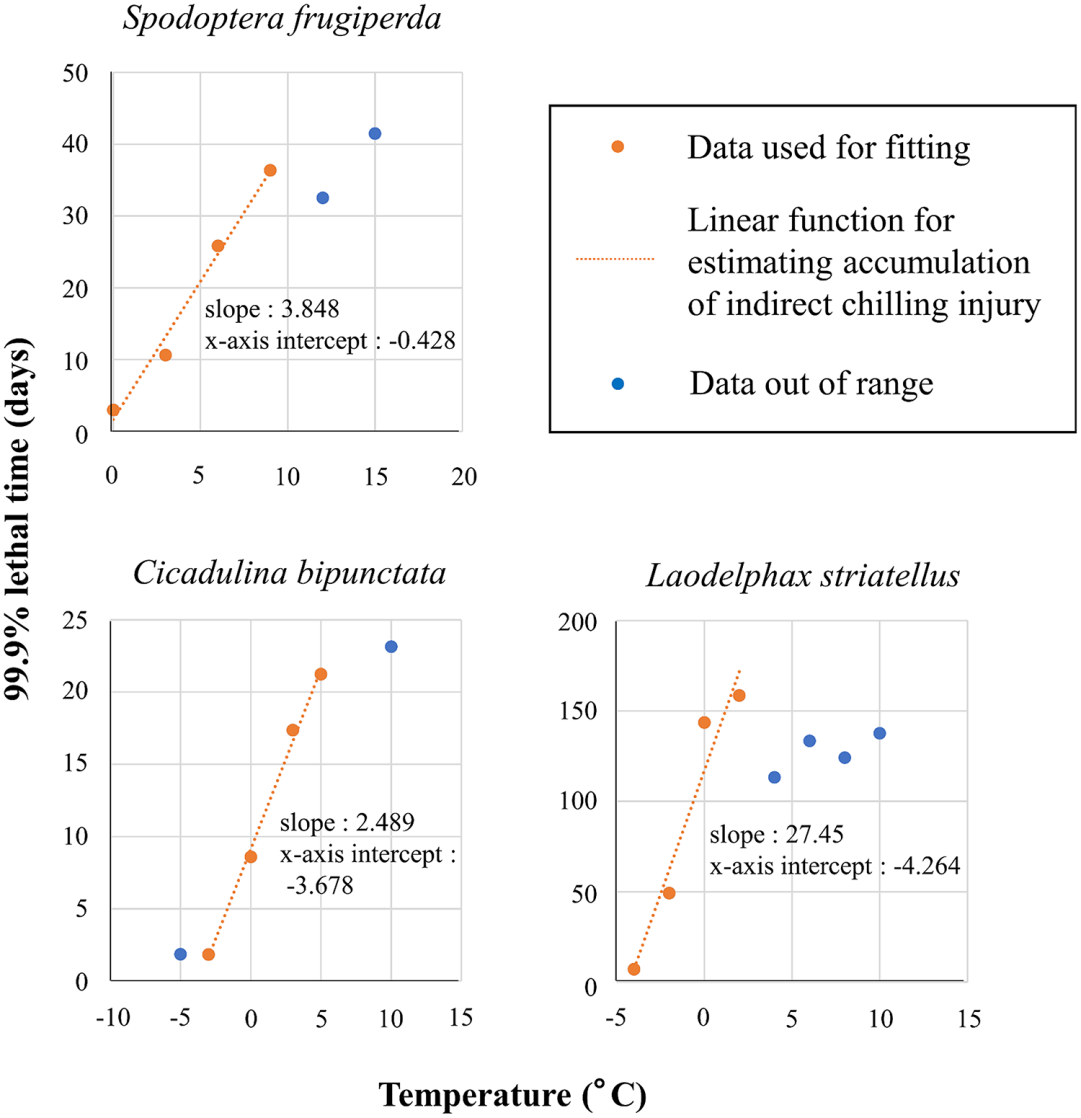
Effects of temperature on the lethal time (i.e., time required for 99.9% mortality, LT_99.9_) in three insect species. Each LT_99.9_ was calculated from published survival data at each temperature by probit analysis. The range of temperatures that showed significance and had the highest *r*^2^ values by linear regression among all temperature ranges was regarded as the range of temperatures at which chilling injury occurred (shown in orange).

The ranges of temperature that showed statistical significance and the highest *r*^2^ values were between 0 and 9 °C for *S. frugiperda* (*P* = 0.007, *r*^2^ = 0.986), between − 3 and 5 °C for *C. bipunctata* (*P* = 0.002, *r*^2^ = 0.996), and between − 4 and 2°C for *L. striatellus* (*P* = 0.033, *r*^2^ = 0.935) (Fig. 1), indicating that chilling injury of each species is proportionally accumulated in these ranges. At temperatures other than these ranges, correlation was not significant (*P* > 0.05) and/or coefficient of determination was lower (Appendix 1)., *Laodelphax striatellus* had a longer LT_99.9_ (e.g., 143.6 days at 0 °C) than *S. frugiperda* (2.973 days at 0 °C) and *C. bipunctata* (8.607 days at 0 °C) (Fig. 1). The lower threshold temperature that chilling damage can be calculated in the fitted linear function (calculated as –*b*/*a* in Eq. 1), were − 0.428 °C for *S. frugiperda*, − 3.678 °C for *C. bipunctata*, and − 4.264 °C for *L. striatellus*.

## Overwintering risks of the three insects based on the developed parameters on chilling injury

**Fig. 2:**
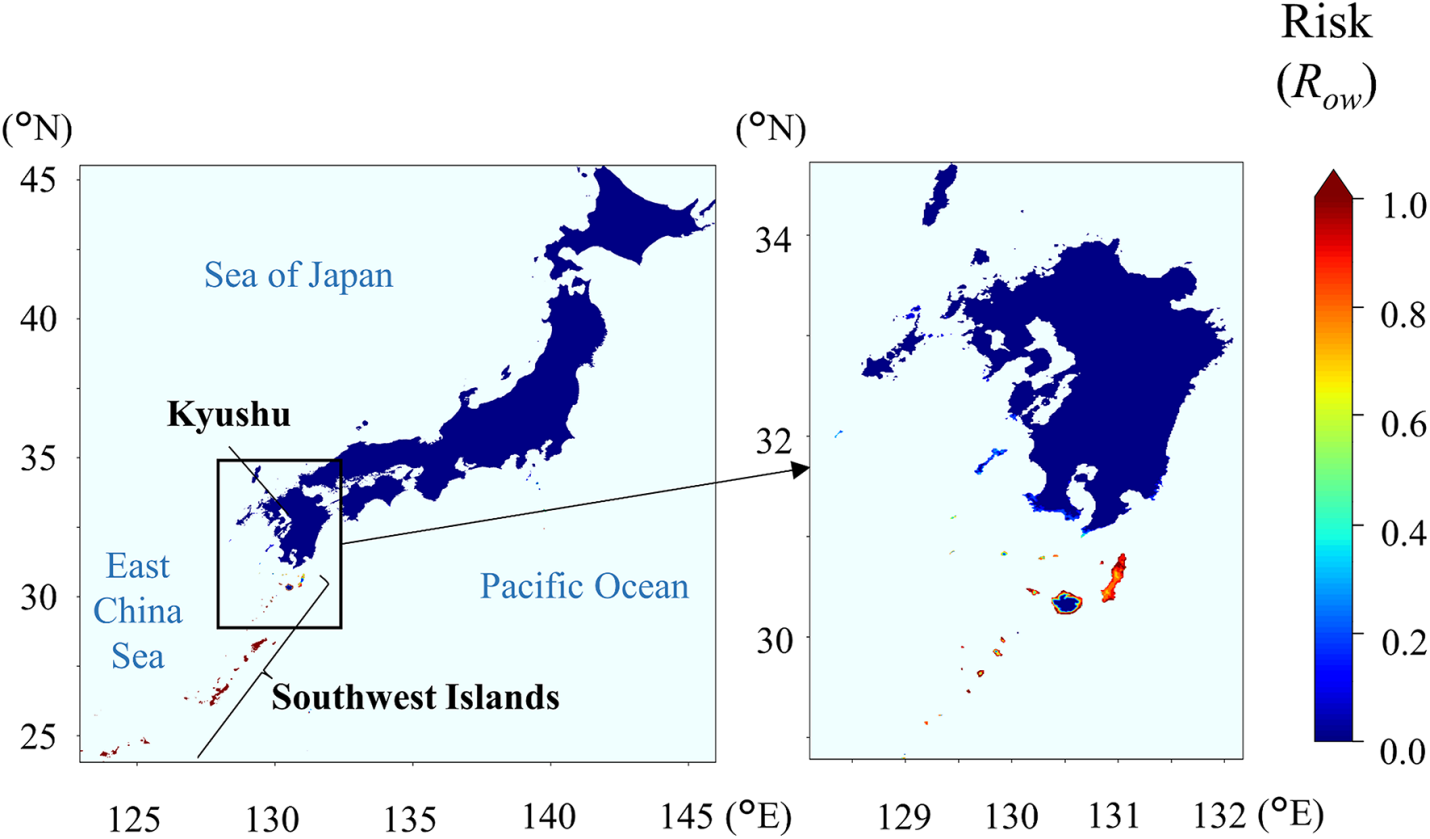
Overwintering risk (*R*_ow_) of *Spodoptera frugiperda*, evaluated by using parameters derived from the time required for 99.9% mortality (LT_99.9_) and past climate data from winter 2010–2011 to winter 2019–2020 for 1-km × 1-km geographic grid squares in Japan. The climate data were obtained from the Agro-Meteorological Grid Square Data System operated by the National Agriculture and Food Research Organization (https://amu.rd.naro.go.jp/wiki_open/doku.php?id=start). High-risk areas (*R*_ow_ > 0.8) were observed only in the Southwest Islands, where overwinter of S. frugiperda was confirmed, and were not found on the main island of Kyushu or in northern areas. (Cartographic software: Python 3.9.10, https://www.python.org/downloads/release/python-3910/).

**Fig. 3:**
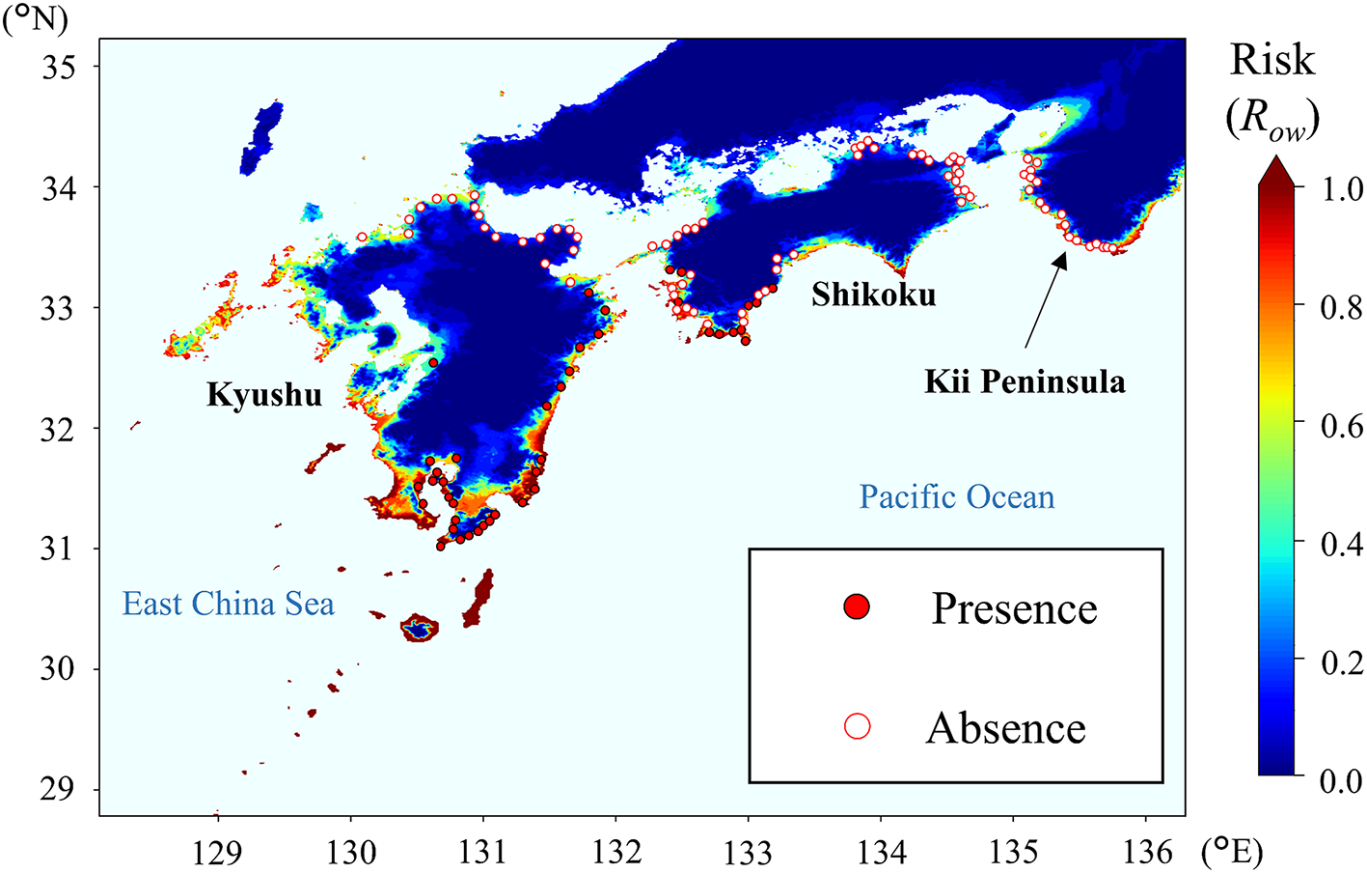
Overwintering risk (*R*_ow_) of *Cicadulina bipunctata*, evaluated by using parameters derived from the time required for 99.9% mortality (LT_99.9_) and past climate data from winter 2000–2001 to winter 2009–2010 for 1-km × 1-km geographic grid squares in Japan. The overwintering risk was 0 in all grids in northern areas that are not shown in the figure. The presence/absence records from 2005 to 2011 (shown as purple circles) are from literatures^32,56^. (Cartographic software: Python 3.9.10, https://www.python.org/downloads/release/python-3910/).

**Fig. 4:**
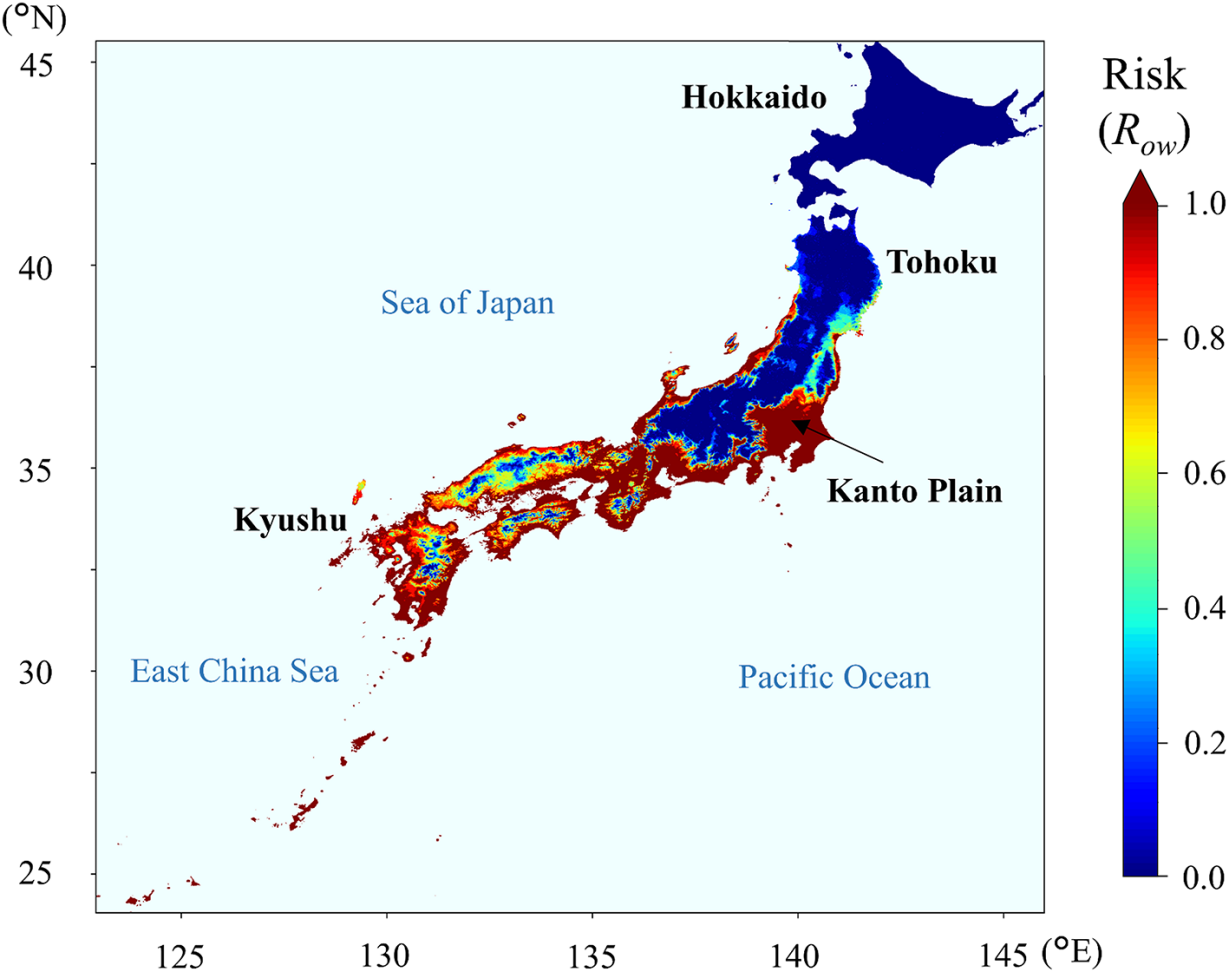
Overwintering risk (*R*_ow_) of *Laodelphax striatellus*, evaluated by using parameters derived from the time required for 99.9% mortality (LT_99.9_) and past climate data from winter 2010–2011 to winter 2019–2020 for 1-km × 1-km geographic grid squares in Japan. Survival data for calculating the LT_99.9_ parameters were obtained by using an overwintering population of *L. striatellus* collected on Kyushu Island in 2017. (Cartographic software: Python 3.9.10, https://www.python.org/downloads/release/python-3910/).

When the overwintering risk (*R*_ow_) of the three insect species in Japan was evaluated based on parameters derived from LT_99.9_, *R*_ow_ of *S. frugiperda* was the lowest among the tested species: the high-risk area (*R*_ow_ > 0.8) was restricted to the Southwest Islands and did not extend to the main island of Kyushu or the northern areas, where the overwintering risk was relatively low (*R*_ow_ < 0.2) at most sites (Fig. 2). The overwintering risk of *C. bipunctata*, as estimated on the basis of winter climate data for the 2000s, was high in a wide range of coastal areas facing the Pacific Ocean and the East China Sea in the Kyushu and Shikoku regions, where this leafhopper is widely distributed, and on the Kii Peninsula, where it is not yet established (Fig. 3). The overwintering risk of *L. striatellus* was greater than 0.8 in most areas of western Japan, except in the highlands, and it was also high on the Kanto Plain and parts of coastal areas in the Tohoku region (Fig. 4). Paradoxically, the risk was low in the northern plain areas of the Tohoku region and the Hokkaido region, where this planthopper is in fact widely distributed^34,35^.

**Observed and simulated mortality dates for*S. frugiperda*during winter**.

A field experiment at the Koshi Research Station in temperate Japan supported the accuracy of developed parameters in evaluating the survival of overwintering adult *S. frugiperda* by our model. The number of days required for mortality of all released adults was 22.3 ± 10.9 days in the 2021 winter season and 15.0 ± 6.90 days in the 2022 winter season (Table 1). Our model estimated the number of days required to kill 99.9% of adults in each replicate to be 10 to 27 days, which varied depending on hourly air temperatures during the experiment. The mean absolute error between the observed and estimated mortality dates was 4.18 days (Table 1). The error tended to be larger (14 days at maximum) in the later replicates in February and March probably because of relatively longer survival duration.

**Table 1:** Estimated and observed dates of mortality of overwintering adult *Spodoptera frugiperda* kept outside.

	Date that adult moths were moved to field	*n*	Estimated date of 99.9% mortality by our model	Date that mortality of all adults was confirmed^†^	Error^‡^ (days)
2021 winter season (Dec 2021–Mar 2022)					
	17 Dec	34	2 Jan	7 Jan	5
	24 Dec	32	3 Jan	7 Jan	4
	31 Dec	33	12 Jan	14 Jan	2
	7 Jan	33	19 Jan	22 Jan	3
	14 Jan	28	1 Feb	31 Jan	–1
	22 Jan	32	18 Feb	25 Feb	7
	22 Jan	33	18 Feb	4 Mar	14
2022 winter season (Dec 2022–Jan 2023)					
	8 Dec	89	29 Dec	2 Jan	4
	17 Dec	52	30 Dec	30 Dec	0
	13 Jan	90	29 Jan	26 Jan	–3
	17 Jan	85	29 Jan	26 Jan	–3
Mean absolute error					4.18

^†^Survival of the adult were checked every few days.

^‡^Number of days calculated as (observed mortality date) – (estimated mortality date).

### Improved discrimination between presence and absence records of*C. bipunctata*by using developed model.

**Fig. 5:**
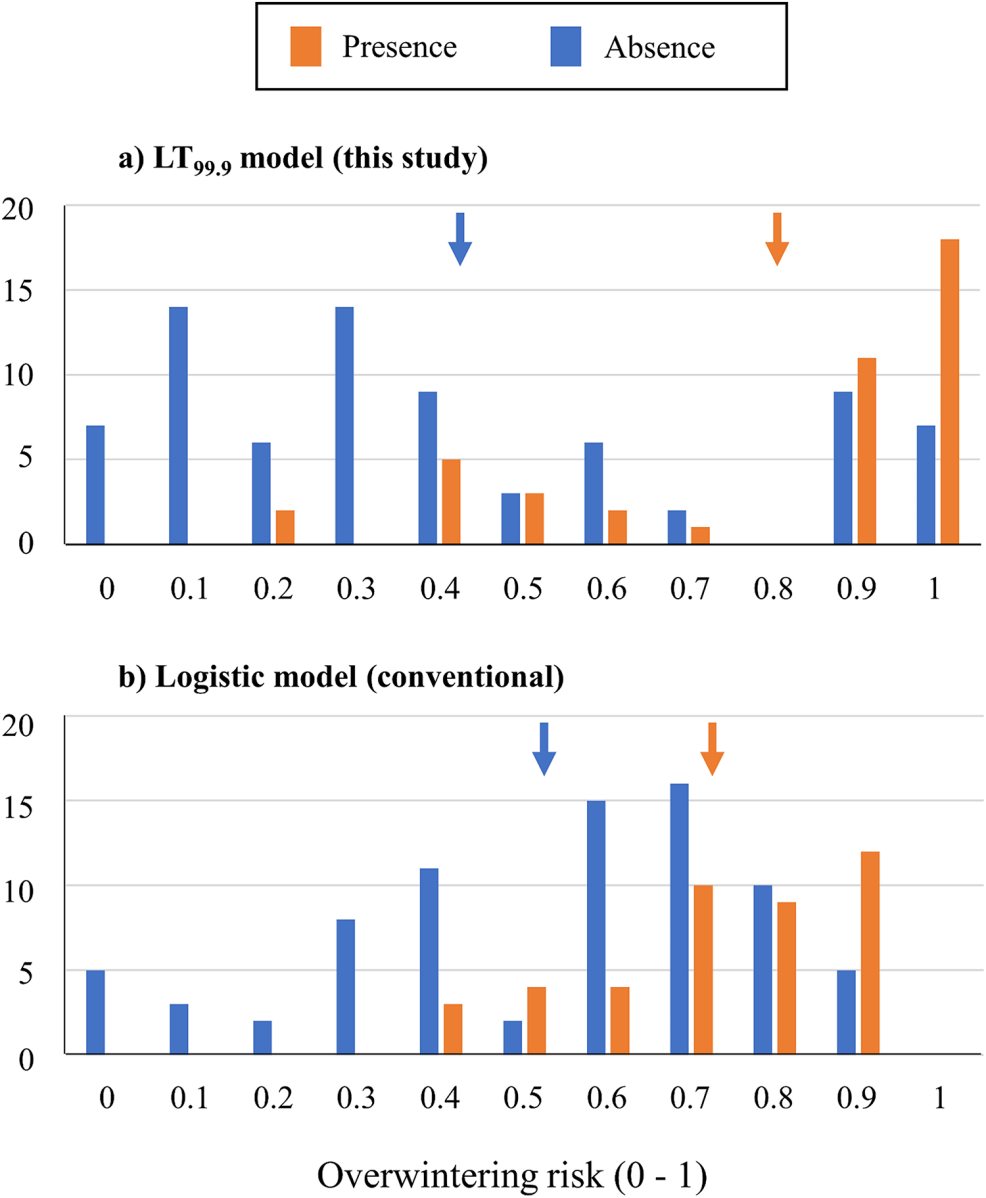
Frequency distributions of overwintering risk (*R*_ow_) estimated for 42 presence and 77 absence records of *Cicadulina bipunctata* by using the present model (**a**) and a conventional logistic model (**b**). Ten years of winter climate data from winter 2000–2001 to winter 2009–2010 at each record site were used to estimate *R*_ow_. Colored arrows indicate the mean *R*_*ow*_ for presence and absence records.

A developed model using parameters of chilling injury discriminated between 42 presence and 77 absence records of *C. bipunctata*better than a conventional logistic model^36^ (Fig. 5). The overwintering risk for absence records estimated by our model (0.416 ± 0.324) was significantly lower than that estimated by the conventional logistic model (0.529 ± 0.245; *P* = 0.039), whereas no significant difference was observed for presence records between the present (0.802 ± 0.257) and the logistic (0.722 ± 0.152) models (*P* = 0.088) (Fig. 5).

## Discussion

This study proposed a novel indicator that quantitatively evaluates the survival of overwintering population of tropical and subtropical insects in temperate regions on the basis of the insects’ tolerance to chilling injury. The overwintering risk map for *S. frugiperda*was consistent with its actual distribution in Japan; this moth rarely becomes localized in most areas, with the exception of the Southwestern Islands^28,37^. This result coincides with the result of published CLIMEX model^38^ that *S. frugiperda* can not establish the population but spent one or more generations during warm season in most regions of Japan. The temporal emergence of *S. frugiperda*during warm season is also known in its native range: the adults annually migrate from subtropical regions of southern North America to northern regions, where they cannot overwinter^39^. After the worldwide range expansion of this moth in the late 2010s, the clear geographical gap between areas suitable for localization (i.e., population establishment) and only for temporal migration (i.e., ephemeral population) for *S. frugiperda*in invaded ranges has been well discussed^9^. These facts support the accuracy of risk evaluation by using the developed indicator. The accuracy of the indicator was also supported by the precise evaluation of overwintering ability in our field experiment. The risk map for *C. bipunctata*based on climate data from 2000 to 2010 coincided with the distribution record of this leafhopper in about 2010^32,40^and also coincided with the risk map generated by the conventional logistic model^36^. Furthermore, the better separation of the overwintering risk of *C. bipunctata* between presence and absence sites in our model than in the conventional logistic model implies that the present model using the developed parameter based on LT_99.9_ is more appropriate for evaluating potential overwintering risk. The results for these two insects of tropical and subtropical origins support our hypothesis that the LT_99.9_ approach using accumulated damage by chilling during winter is effective in evaluating the overwintering risk of insect pests of tropical and subtropical origins in temperate regions.

The new model using damage by chilling as a parameter has an advantage in that it can be used for the quick risk assessment of newly emerging invasive pests from tropical and subtropical regions. Generally, invasive species that have serious impacts on indigenous ecosystems reproduce well and rapidly expand their populations in the invaded range by their ability to overcome selective pressures from climate, food, and natural enemies in the invaded range^41–43^. Control of invasive crop pests at the early stage of their invasion is therefore important to minimize negative impacts on crop production in indigenous agroecosystems. The LT under moderately low temperatures—the only parameter required to run our model—is not determined from field data but can be obtained in the laboratory. The easy and quick determination of parameters in our model makes it appropriate for use in the first, or preliminary, risk evaluation of overwinter of newly invading, or potentially invading, species in temperate regions.

Although our model has practical application to insect pests from tropical and subtropical regions, it should be applied carefully to insects that have previously been distributed in temperate regions. The distribution ranges of *S. frugiperda* and *C. bipunctata* estimated from our model reasonably reflected their actual distributions, but the results for *L. striatellus*were not accurate, particularly in the northern areas, namely Hokkaido and Tohoku, where this planthopper is widely distributed on the plains^35^. Geographical divergence of the insect’s populations might have influenced this prediction. Despite the annual long-distance migration of *L. striatellus*from south to north in East Asia^34^, some evidence suggests the existence of geographical variations in this planthopper, such as allozyme polymorphism^44^, variations in susceptibility to insecticides^45^, the presence of endosymbionts^46^, and variations in the phylogeny of transmitted viruses^47^. Furthermore, this planthopper is widely distributed in Eurasia including subarctic regions such as Norway, Finland, and Russia^48^. If local populations have diverged tolerance to low temperatures as the result of adaptation to the environment (e.g., average temperature during overwinter, annual minimum temperature, and duration of overwinter season) of each habitat, then the use of a single parameter value determined from a specific geographical population will lead to misevaluation of the overwintering risk.

The correlative relationship between LT_99.9_and exposure temperature was highly significant in all three insect species tested. This result coincides with the physiological fact that chilling damage is gradually accumulated in insects’ bodies through physiological stress^21,22,26^. However, the range of temperatures that gave the highest *r*^2^values differed among the species, probably because of interspecies variations in tolerance to low temperatures. Previous studies using the time–temperature model^49^ have reported that the upper threshold temperature causing chilling injury is 15 °C in *S. frugiperda*, 5 °C in *C. bipunctata*, and 0 °C in *L. striatellus*^50,51^, whereas our current linear regression analysis estimated the respective temperatures to be 9 °C, 5 °C, and 2 °C. Although the variation of the upper threshold temperatures in *S. frugiperda* and *L. striatellus* had little effect on the risk evaluation because of no significant difference in changes of survival curves at temperatures between 9 and 15 °C in *S. frugiperda*^51^ and between 2 and 0 °C in *L. striatellus*^35^, the threshold temperatures should be determined objectively in such modelling approaches. The time–temperature model can mathematically (i.e., objectively according to statistical values) determine the upper threshold temperature, but it requires modification of the calculation of cumulative chilling damage (Eq. 2) to a non-linear one. To improve the accuracy and clarify the generality of the new model, further mathematical approaches to determining the target temperature range are needed. Regarding better determination of the upper threshold temperature, quantifying chilling damage in insects’ body such as disruption of osmoregulation, changes in membrane phase, and loss of ion homeostasis^26,52^ are also effective.

Expanding the model to evaluate overwintering risk over multiple developmental stages is another direction to lead more accurate prediction. This is not mandatory for insect species that overwinter in particular developmental stage such as *C. bipunctata* and *L. striatellus*, but is important for insects that the overwintering stage is uncertain. In this study, we preliminarily confirmed that survival data of adult is the most appropriate to evaluate the overwintering risk for *S. frugiperda* in our model. This may have a risk of underestimation of the overwintering ability of this new invasive pest. All adults will die regardless of their tolerance to low temperature, therefore, determined LT_99.9_from adult survival data never exceeds the adult lifespan. On the other hand, larva and pupa can proceed to next developmental stage if they can survive and develop under exposed low temperatures. In fact, survival curves of mature larva at 9 °C and higher temperatures do not significantly differ from those of adult although mature larva is intolerant to severer low temperatures from 3 °C to 6 °C^44^. Considering the survival and development of larva and pupa as well as adult during winter may be needed to update the overwintering risk map of *S. frugiperda* shown in this study.

The risk of novel invasion of tropical and subtropical insect pests into temperate regions is increasing with global warming. Here, we found a proportional change in LT_99.9_ under constant low temperatures causing chilling injury, and we confirmed that an indicator based on LT_99.9_can be used with high accuracies to estimate the overwintering risks of two insects of tropical and subtropical origins in temperate regions by SDM approach. For insect pests for which the winter climate strictly restricts survival in temperate regions, this indicator can be used as an intuitive determinant of niche availability, in much the same way as the supercooling point is used for organisms in cold regions^53–55^. Further studies using other insect pests should confirm the validity and generality of the indicator.

## Materials and methods

### Survival data under low temperatures

Survival data for the three insect species under moderately low temperatures were obtained from established reports^35,50,51^. Through the examination of survival rates every few days or weekly, these reports present survival curves for each insect species under constant low-temperature conditions at which chilling injury occurs. We used the survival data of adult *S. frugiperda* (six temperature conditions from 0 to 15 °C, *n* = 22 to 31 for each condition) for model construction because we preliminarily confirmed that adult can survive lower temperature than larva and pupa (Appendix 2). The adult survival data was also used for *C. bipunctata* (six temperature conditions from − 5 to 10 °C, *n*= 100 for each condition), because this leafhopper overwinters as an adult^56^. The adults of *S. frugiperda* and *C. bipunctata* used in these previous reports were laboratory reared strains originally collected in Kumamoto Prefecture, Japan. On the other hand, we adopted nymphal survival data for *L. striatellus* (eight temperature conditions from − 4 to 10 °C, *n*= 100 for each condition), because this insect overwinters at the 3rd and 4th instar nymph^57^. Overwintering nymphs collected in Kumamoto Prefecture, Japan were used to obtain the survival data. Ranges of the upper-limit temperatures at which substantial decreases in the survival period due to chilling injury appears are 9 to 15 °C in *S. frugiperda;* 3 to 5 °C in *C. bipunctata*; and − 2 to 0 °C in *L. striatellus*^43,44,58^. These insects do not freeze at temperatures above − 10 °C.

### Linear regression of time required for 99.9% mortality at constant low temperatures

We adopted LT_99.9_ as an indicator of the overwintering success of the target populations, because it is impossible to determine mathematically the time and temperature conditions that kill 100% of individuals. This indicator predicts the overwintering success or failure of insects as population units (i.e., not individual survival) on the basis of the accumulation of chilling injury during overwintering. From the survival data of each insect species, LT_99.9_ at each temperature was determined by probit analysis. We then used linear regression analysis to examine the correlation between temperature and LT_99.9_, as given by the following equation:

1 $$\:{P}_{T}=aT+b$$

where *P*_*T*_ is the minimum period of time required to kill 99.9% of insects exposed to the constant temperature *T*, and *a* and *b* are parameters to be estimated. Because the survival data from the established papers contained data out of the temperature range that caused chilling injury, for model construction we chose a range of temperatures showing significance (*P* < 0.05, Pearson’s product moment correlation) and the highest coefficient of determination (*r*^2^) between *T* and *P*_*T*_.

## Accumulation of chilling damage under fluctuated temperature conditions

In order to evaluate the overwintering risk from the species-specific parameters *a* and *b* in Eq. 1, cumulative damage from chilling, *D*_cum_, within the targeted temperature range was calculated as follows:

2 $$\:{D}_{\text{c}\text{u}\text{m}}=\sum\:\frac{1}{{P}_{T}}$$

In this equation, *D*_cum_ was indexed as the sum of injury per unit time (*P*_*T*_^[–1^) at temperature *T*. When *D*_cum_ reaches 1 or higher, no insect (actually fewer than 0.1% in this study) is regarded as alive. Note that values of *D*_cum_ < 1 do not reflect an overwintering risk, such as the probability of overwintering or the survival rate of individuals during winter, because the model considers only the temperature conditions that cause 99.9% mortality of the population.

For *C. bipunctata* and *L. striatellus*, *P*_*T*_ was calculated at daily unit from Eq. 1 by substituting the daily average temperature to *T*. On the other hand, *P*_*T*_ for *S. frugiperda* was calculated at hourly unit by using Eq. 3. This was used because *S. frugiperda* undergoes mitigation of damage from chilling injury when exposed to temperatures of 17.5 °C or higher for at least 2 h/day^51^.

3 $$\:{D}_{\text{c}\text{u}\text{m}}=\sum\:_{d}\sum\:_{h}^{24}\frac{{M}_{d}}{24\:\times\:\:{P}_{T}}$$

This equation calculates *D*_cum_ as the sum of hourly (*h*) damage from chilling injury, which can be calculated as *P*_*T*_ divided by 24. Four values for the coefficient of repair *M*_*d*_ for each day *d*were determined in accordance with the recovery rate reported by Tanaka and Matsukura^44^. We determined *M*_*d*_ = 1 when there were fewer than two hourly temperatures higher than 17.5 °C during the day; *M*_*d*_ = 0.379 if hourly temperatures between 17.5 and 25 °C lasted for 2 h or longer; *M*_*d*_ = 0.261 when one hourly temperature was higher than 25 °C and there was at least 1 h of temperatures between 17.5 and 25 °C; and *M*_*d*_ = 0.143 if hourly temperatures higher than 25 °C lasted 2 h or more.

For all three insects, *P*_*T*_ was regarded as 0 when the daily or hourly temperature was higher than the upper threshold temperature causing chilling injury. When grid squares contained daily average temperatures lower than the range of temperatures causing chilling injury, *P*_*T*_ was fixed at 1 and 1/24 in daily and hourly calculations, respectively. The overwintering risk from December to March was evaluated for each 1 km × 1 km geographical grid square of entire Japan. The daily mean temperature in each geographical grid square (383,636 grid squares in total) was obtained from the Agro-Meteorological Grid Square Data System operated by the National Agriculture and Food Research Organization (https://amu.rd.naro.go.jp/wiki_open/doku.php?id=start). Hourly temperature data used for evaluation of *S. frugiperda*were disaggregated from the maximum and minimum temperatures and daily daylength obtained from the Agro-Meteorological Grid Square Data System by using the modified sine curve model^59^. The two coefficients of the modified sine curve model (parameters *a* and *b*of the model^59^) were preliminarily optimized to be 1 and 0.5.

## Generating maps showing overwintering risks of the three insects

We used 10 years’ climate data to evaluate the overwintering risk of each insect species. By using multiple years’ data, we could consider annual fluctuations of winter temperatures and evaluate the overwintering risk quantitatively because our model outputs binary result (overwintered or not) to one seasonal climate data. To compare the estimated overwintering risks with the latest distribution records of each species, past climate data from winter 2010–2011 to winter 2019–2020 were used for *S. frugiperda* and *L. striatellus*. On the other hand, data from winter 2000–2001 to winter 2009–2010 were used for *C. bipunctata* because no distribution record newer than 2012 was available. After calculating *D*_cum_ for each year and grid square, we defined the overwintering risk (*R*_ow_) in the grid square as the ratio of the number of years in which *D*_cum_ was < 1 to the total number of years tested (*n* = 10). Unlike *D*_cum_, *R*_ow_ is a quantitative index proportionally reflecting the overwintering risk.

### Field verification of determined parameters in *S. frugiperda.*

The validity of the minimum period of time required for population mortality (*P*_*T*_) was examined by a field test in the experimental field at Koshi Research Station (32°52′43ʺN, 130°44′25″E). We used a laboratory-reared strain of *S. frugiperda* that were originally collected from a forage maize field at the same research station in May and August 2020 and then maintained at 25 °C on an artificial diet (Insecta LFS; Nosan Corp., Kanagawa, Japan). Newly emerged adults (male and female mixed) were placed in a plastic cup (150-mm diameter × 80-mm height) with several layers of nonwoven fabric (20 mm × 60 mm) for hibernation and honey solution as food. They were then exposed to temperatures of 15 °C for 7 to 10 days to acclimatize them to the cold. After cold acclimation of the insects, the plastic cups were placed in an acrylic box (340 mm long × 260 mm wide × 340 mm high; fine nylon mesh on three sides for ventilation; Sanshin Industrial Co., Ltd., Kanagawa, Japan) to prevent the adults from escaping during the field test because *S. frugiperda* was a novel invasive crop pest in Japan. The acrylic box was then placed in the experimental field. Hourly temperature in the acrylic box was monitored by a data logger (TR72A; T&D Corp., Nagano, Japan). The experiments were performed seven times in the 2021 winter season, from December 2021 to March 2022, and four times in the 2022 winter season, from December 2022 to January 2023. During the overwintering experiment, the number of dead adults was counted once per week in the 2021 winter season and every two to three days in the 2022 winter season. For each treatment we used 28–34 adults (93 females and 132 males) in the 2021 winter season and 52–90 adults (165 females and 151 males) in the 2022 winter season. The mortality dates were estimated from the hourly temperature monitoring data from this field experiment by using our model (Eq. 3). The estimated dates of 99.9% mortality were compared with the date when the field observations confirmed the death of all adults tested.

### Accuracy of risk evaluation compared with that using a conventional mechanistic model for *C. bipunctata*

The model accuracy was compared between our model and a conventional logistic model of *C. bipunctata*that we had developed previously^36^. Briefly, the conventional model evaluates the probability of distribution, *P*_dist_, by using the following logistic function:

4 $$\:{P}_{\text{d}\text{i}\text{s}\text{t}}=\frac{1}{1+{e}^{0.05{T}_{\text{c}\text{u}\text{m}}-3.07}}$$

where *T*_cum_ is the cumulative daily low temperature under 5 °C during winter. We compared the accuracy between our new model and the conventional logistic model by using 42 presence and 77 absence records of *C. bipunctata* in 2011 and 2012^32,44^. In these reports, the planthopper is judged to be present when 25–30 times sweeping on gramineous plants in autumn with an insect net (42 cm in diameter) caught one or more *C. bipunctata* whereas areas where no *C. bipunctata* was collected by the sweeping were regarded to be absent data. For each record, *R*_ow_ and *P*_dist_ were calculated from our present model and the conventional model, respectively, on the basis of 10 years of winter climate data from winter 2000–2001 to winter 2009–2010 obtained from the Agro-Meteorological Grid Square Data System.

### Simulation and data processing

The new model was executed and verified by using Python ver. 3.8 or newer on a personal computer in our laboratory and on the “Shiho” supercomputer operated by the National Agriculture and Food Research Organization of Japan.

## Electronic supplementary material

Below is the link to the electronic supplementary material.

Supplementary Material 1

Supplementary Material 2

## Data Availability

Probit-transformed survival data for the three insect species tested, hourly temperature data during the field tests, hourly winter temperatures (December to March) from 2011 to 2012 to 2020–2021 in 10 cities in Japan, provided by the Japan Meteorological Agency, and Python codes used to draw the overwintering risk map of each insect species are available from figshare: https://figshare.com/s/9a6e39ec2ea034f51b02 Note that this is currently a private link only for peer review. It will be updated to a permanent link with DOI after the manuscript is accepted for publication.
