# Supplementary material for: Evaluation of overwintering risk of tropical and subtropical insect pests in temperate regions
Source: Sci Rep. 2024 Dec 28;14:31333. doi: 10.1038/s41598-024-82713-z (PMC11682330; doi:10.1038/s41598-024-82713-z)
Supplement: Supplementary file 1 — Supplementary Material 1 [file 41598_2024_82713_MOESM1_ESM.pdf]

Appendix 1

Evaluation of overwintering risk of tropical and subtropical insect pests in temperate regions

Table 1 Coefficient of determination (r^2), statistical significance (P), and coefficients (a and b) of linear function fitted to time required to kill 99.9% individuals of insect population in each range of temperature.

Species	Range of temperature (°C)		r^2	$P^†$	Coefficients of fitted linear function‡	
	Lower	Upper			a	b
Spodoptera frugiperda						
	0	15	0.897	0.004	2.561	5.774
	0	12	0.875	0.020	2.829	4.701
	**0**	**9**	**0.986**	**0.007**	**3.848**	**1.645**
	0	6	0.965	0.076	3.814	1.713
	3	15	0.822	0.034	2.281	8.856
	3	12	0.753	0.132	2.541	7.293
	3	9	0.989	0.067	4.291	-1.454
	6	15	0.712	0.156	1.437	18.983
	6	12	0.394	0.568	1.114	21.567
	9	15	0.325	0.560	0.855	26.555
Cicadulina bipunctata						
	-5	10	0.918	0.003	1.662	9.577
	-5	5	0.960	0.003	2.109	10.185
	-5	3	0.927	0.037	2.022	9.948
	-5	0	0.840	0.262	1.419	7.891
	-3	10	0.887	0.017	1.711	9.309
	**-3**	**5**	**0.996**	**0.002**	**2.489**	**9.154**
	-3	3	0.995	0.047	2.586	9.271
	0	10	0.808	0.101	1.381	11.378
	0	5	0.989	0.066	2.558	8.916
	3	10	0.821	0.278	0.742	16.138
Laodelphax striatellus						
	-4	10	0.455	0.067	7.228	86.722
	-4	8	0.468	0.090	8.745	86.722
	-4	6	0.564	0.085	11.997	88.890
	-4	4	0.625	0.111	16.095	94.354
	**-4**	**2**	**0.935**	**0.033**	**27.453**	**117.069**
	-4	0	0.954	0.138	34.153	134.937

-2	10	0.192	0.325	3.599	108.495
-2	8	0.175	0.409	4.271	107.599
-2	6	0.263	0.377	6.912	105.839
-2	4	0.305	0.448	10.353	105.839
-2	2	0.850	0.253	27.326	117.154
0	10	0.147	-	-1.603	143.180
0	8	0.335	-	-3.196	147.428
0	6	0.263	-	-3.773	148.582
0	4	0.432	-	-7.578	153.654
2	10	0.083	-	-1.538	142.699
2	8	0.308	-	-4.149	153.145
2	6	0.304	-	-6.256	160.167
4	10	0.578	0.240	3.200	104.799
4	8	0.287	0.640	2.714	107.388
6	10	0.092	0.804	1.054	123.393

†: Statistical significance was evaluated by Pearson's product moment correlation.

‡: *a* and *b* represent slope and intercept of the fitted function, respectively.
