# Supplementary material for: Evaluation of overwintering risk of tropical and subtropical insect pests in temperate regions
Source: Sci Rep. 2024 Dec 28;14:31333. doi: 10.1038/s41598-024-82713-z (PMC11682330; doi:10.1038/s41598-024-82713-z)
Supplement: Supplementary file 2 — Supplementary Material 2 [file 41598_2024_82713_MOESM2_ESM.pdf]

Appendix 2

Evaluation of overwintering risk of tropical and subtropical insect pests in temperate regions

Table A2-1 Coefficient of determination (r^2), statistical significance (P), and coefficients (a and b) of linear function fitted to time required to kill 99.9% individuals of matured larva, female pupa, and adult of *Spodoptera frugiperda* in each range of temperature.

Stage	Range of temperature (°C)		r^2	$P^†$	Coefficients of fitted linear function‡	
	Lower	Upper			a	b
Matured larva						
	6	15	0.642	0.313	215.430	-1645.082
	**6**	**12**	**0.995**	**0.045**	**15.257**	**-67.697**
	9	15	0.772	0.317	347.618	-3363.530
Female pupa						
	3	15	0.994	<0.001	9.485	-26.165
	3	12	0.988	0.006	9.350	-25.357
	3	9	0.972	0.108	9.428	-25.747
	6	15	0.994	0.003	9.991	-32.238
	6	12	0.988	0.071	10.228	-34.135
	**9**	**15**	**0.997**	**0.037**	**9.170**	**-21.562**
Adult						
	0	15	0.897	0.004	2.561	5.774
	0	12	0.875	0.020	2.829	4.701
	**0**	**9**	**0.986**	**0.007**	**3.848**	**1.645**
	0	6	0.965	0.076	3.814	1.713
	3	15	0.822	0.034	2.281	8.856
	3	12	0.753	0.132	2.541	7.293
	3	9	0.989	0.067	4.291	-1.454
	6	15	0.712	0.156	1.437	18.983
	6	12	0.394	0.568	1.114	21.567
	9	15	0.325	0.560	0.855	26.555

†: Statistical significance was evaluated by Pearson's product moment correlation.

‡: *a* and *b* represent slope and intercept of the fitted function, respectively.

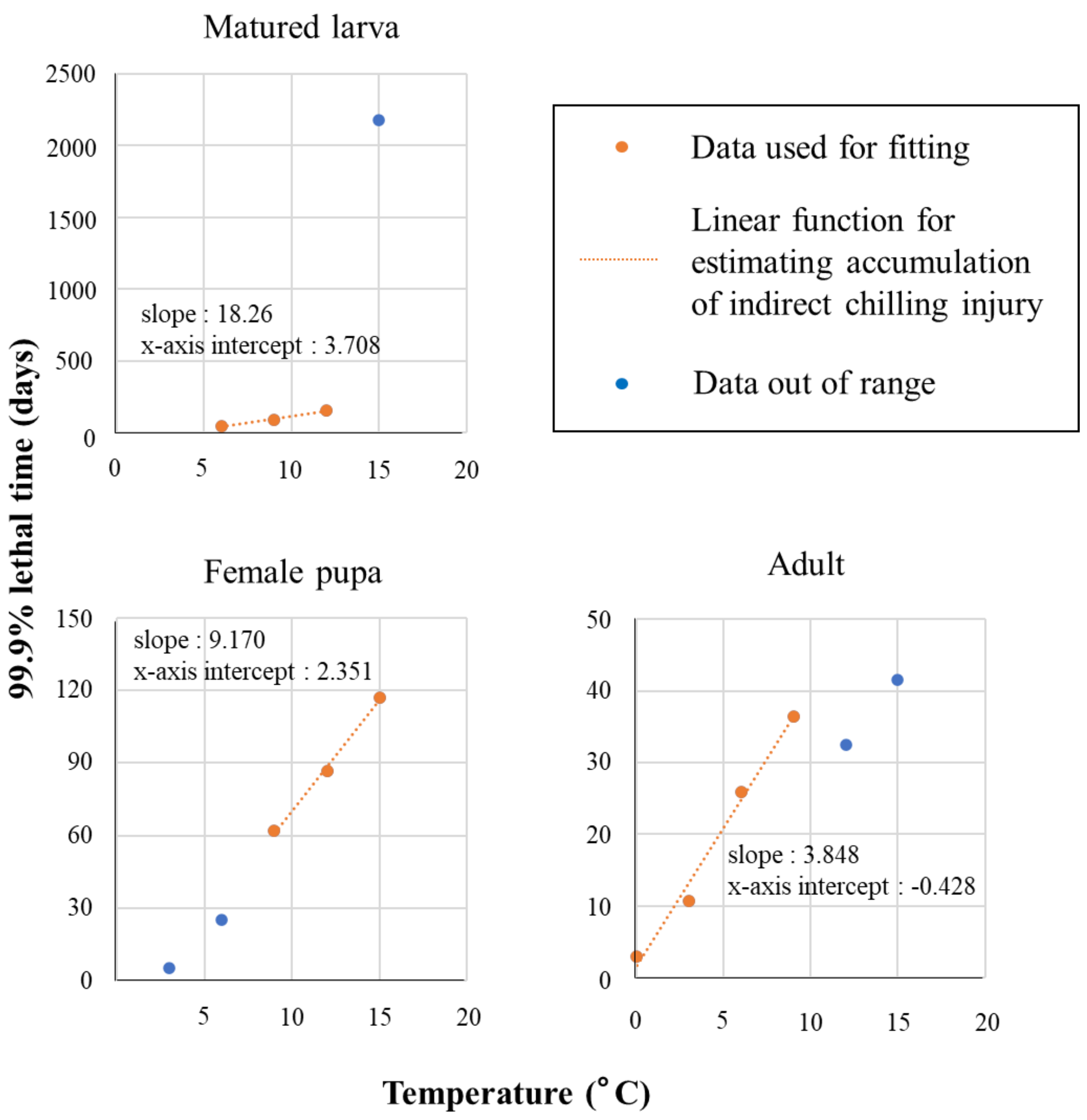

Figure A2-1 Effects of temperature on the lethal time (i.e., time required for 99.9% mortality, $LT_{99.9}$) in three developmental stages of *Spodoptera frugiperda*. Each $LT_{99.9}$ was calculated from published survival data at each temperature by probit analysis. The range of temperatures that showed significance and had the highest r^2 values by linear regression among all temperature ranges was regarded as the range of temperatures at which chilling injury occurred (shown in orange). The values of x-axis intercept indicate that larva and pupa can not survive at temperatures below ~3.7°C and ~2.4°C, respectively, whereas adult can survive even at ~-0.4 °C.
